# Case of Colloid Degeneration of Right Lung

**Published:** 1850-09

**Authors:** P. A. Allaire

**Affiliations:** Aurora Illinois


### ARTICLE I.

*Case of Colloid Degeneration of right Lung.* By P. A. ALLAIRE, M. D. Aurora Illinois.

The following case of Malignant Degeneration of the lung will, I think be found of interest from its rareness, and from the difficulty of diagnosis which usually attends cases of a kindred character. Dr. Stokes published a case some years since in the "Dublin Journal of Medical Science," of "Encephaloid Degeneration of the lung," and remarks that it is the "first case in which a direct diagnosis had been made" of the disease in that locality. I have seen no report of a like case since his. It may be found quoted in No. 24 of the New York Medical Gazette, Vol. 2nd.

CASE.—Mr. Windit applied to me in March 1847, for advice in the treatment of a tumor growing on the right foot. On examination I found a growth the size of a hens egg, attached to the metatarsal bone of the great toe, on its upper or dorsal surface. Tumor immovable and without pain; no enlarged vessels on its surface. The part had never received any injury. This young man (Aug. 21.) presented every appearance of perfect health, was a farmer, temperate, and laborious, and his family were free from any hereditary disease.

I had treated Mr. W. about six years before for symptoms of Morbus Coxarius of right hip, the recovery from which had been perfect, except leaving the movements of the joint somewhat abridged, the effect of which was quite apparent in his gait.

On his requesting of me a prescription for the tumor, I expressed to him my fears that the disease was of a cancerous character, and advised its immediate removal, as giving him his only chance for ultimate safety. To this he would not consent, but declared he would wait, watch it, and try for its removal without operation.

May 8th. Was called to see Mr. W., and found the tumor much inflamed in consequence of an injury; he informed me it had steadily increased in size since March. I again proposed the necessity of its removal, which was now agreed to by the patient, but deferred for a few days to abate the local inflammation and prepare him for the operation.

May 11th. Assisted by Drs. Eastman and Stevens I amputated the foot by Chopart's method, which, while it left a very useful stump, was deemed the safest, as removing not only the disease, but the parts immediately adjacent. The tumor, on examination after its removal, was found to consist of a soft degeneration. The medullary part of the first metatarsal bone being filled with a substance resembling brain softened by decomposition. This was the centre of the disease: its bony sides were expanded, thinned, and perforated. Surrounding the bone was a semi-transparent, jelly-like substance, which, as you approached the surface of the tumor, became more like scirrhus, and then gradually the evidences of malignancy were lost in healthy structure. From this dissection, it seemed proper to class the disease as *Colloid*, or *Pultaceous Cancer*.

June 20th. The stump has healed. The patient seems well in every particular and has a very useful limb; in fact the utility of the member seems scarcely abridged.

After this, no interruption of the general good health of Mr. W., occurred for nearly two years, during which time he continued his simple habits and active life, and, from his excellent constitution and the thorough removal of the disease, I had begun to believe that here we had an evidence of the utility of the early removal of malignant growths. But the event proved in this instance, as in most, if not all others of *real* malignancy, that the removal of the point where morbific matter had been deposited, or developed, caused no cessation of the action which first produced it. Ought we indeed to expect, that a mere amputation would produce so radical a change? For none of this class of diseases can be thought local in their character; and when one point of malignancy has been removed, we must look for the development of another. If this is so, the rational plan would be to operate merely for the prolongation of life, and seek diligently for some agent which may remove this diathesis, or act as an antidote to the poison as it is generated.

May 7th. 1849. Saw Mr. W., at his request, and learned from him that he had had a cough, with difficulty of breathing, for nearly two months; these symptoms, slight at first, had gradually increased; the expectoration had been during this time a frothy mucus.

*Present Condition.* Orthopnoea, frequent, short hacking cough, mucous sputa, slightly tenacious and often streaked with bright blood, no pain in the chest, except a little low on the left side, pulse 85, rather small, skin moist, bowels regular, alvine evacuations natural in appearance, tongue clean, appetite good. A little exertion increases the respiratory movements. Percussion gives a dull sound on right side, except immediately under the clavicle, left side resonant throughout. Auscultation gives no respiratory murmur or other sound for the right lung, except in the superior fifth, and there it is mixed with mucous rhoncus. On left side respiratory murmur puerile, in the lower fourth, with crepitous rhoncus. The measurements of the two sides correspond. The symptoms and signs detailed indicated solidification of right lung, or empyema of right side of the chest, or a plueritic effusion in that part. I inclined to the first idea, and expressed the opinion that the solidification was owing to the developement of malignant disease, because 1st There had been no pain or other symptoms of inflammatory action, which must have preceded either empyema, or plueritic effusion, 2nd. from the total impermeability of the lung except the upper portion. 3rd. from the slow but steady increase of orthopnœa, and 4th. from the previous existence of malignant disease. The left lung was evidently sound, except a slight inflammation in its lower portion. I prescribed for the inflammation, a solution Tart. Ant. et. Potass. with poultices to the left side.

May 12th. Symptoms of pneumonia subsided, no change in other respects. Directed Anodynes "*pro re nata*," stimulating expectorants, and mercury as an alterative, hoping good from it if the disease was not malignant, feeling that anything would be useless if it was.

June 1. The patient has been under the influence of the mercurial for ten days, but without any amelioration of symptoms; on the contrary the difficulty of respiration has steadily increased. The right side is now dull on percussion throughout, nor does auscultation now give any sign that air enters the right lung or even its bronchia. Examined the heart, and found it performing its functions healthily. Since last report he has been bled twice, in conequence of the lung being apparently unable to supply the requisite amount of oxygen to the blood, as was indicated by lividity of lips, tips of fingers &c., and by increased embarrassment of respiration. The expectoration is at times very copious, consisting of mucus, marked occasionally with bright blood or little clots. The appetite has failed. Pulse 90 and small. Patient emaciating fast. No effusion in abdomen or cellular tissue. Size of chest same on both sides. Directed to continue anodynes and expectorants, suspend the use of the Calomel.

June 15. Respiration so difficult that he sits up constantly, and has some one to fan him day and night. In the left lung there is now cavernous rhoncus, with a puffing noise in the upper half, in the lower half mucus rhoncus with puerile murmur. No change in right side. These signs of engorgement and abscess in the left lung, show that it will not much longer suffice to keep the vital spark burning. He is using anodynes tonics and stimulants.

June 28th. Died without apparent change in symptoms. About three weeks before his death a small tumor was noticed in the right groin which increased to the size of a pullet's egg. Twenty eight hours after death the body was examined by Dr. N. Hard and myself. In consequence of the heat of the weather and other circumstances the chest only was opened. Body much emaciated, no infiltration of cellular tissue. On opening the thorax, fluid of a pale straw colour; limpid, and without flakes, flowed out to the amount of 18 or 20 ounces, from the right side chiefly. *No trace of lung or bronchial tube could be found on this side, but in the upper part was impacted, and adherent to the parietes, an amber coloured and jelly like mass,* which would probably have weighed six or seven pounds. The lower part of the cavity was occupied with the fluid before mentioned. Left lung adherent to *pleura costalis* in several places, by old bands chiefly; in the upper half were several small cavities filled with grumous fluid. The remainder of this organ was much engorged and softened. The tumor in the groin on being cut into discharged a limpid fluid; but at the centre had the appearance of Scirrhus.

